# Climate change adaptation needs a science of culture

**DOI:** 10.1098/rstb.2022.0390

**Published:** 2023-11-06

**Authors:** Anne Pisor, J. Stephen Lansing, Kate Magargal

**Affiliations:** ^1^ Department of Anthropology, Washington State University, Pullman, WA 99164-1009, USA; ^2^ Department of Human Behavior, Ecology and Culture, Max Planck Institute for Evolutionary Anthropology, 04103 Leipzig, Sachsen, Germany; ^3^ Santa Fe Institute, NM87506, USA; ^4^ Complexity Science Hub, 1080 Vienna, Austria; ^5^ Environmental and Sustainability Studies, University of Utah Health, Salt Lake City, UT84112, USA

**Keywords:** climate change, culture, cultural evolution, adaptation, climate change adaptation

## Abstract

There is global consensus that we must immediately prioritize climate change adaptation—change in response to or anticipation of risks from climate change. Some researchers and policymakers urge ‘transformative change’, a complete break from past practices, yet report having little data on whether new practices reduce the risks communities face, even over the short term. However, researchers have some leads: human communities have long generated solutions to changing climate, and scientists who study culture have examples of effective and persistent solutions. This theme issue discusses cultural adaptation to climate change, and in this paper, we review how processes of biological adaptation, including innovation, modification, selective retention and transmission, shape the landscapes decision-makers care about—from which solutions emerge in communities, to the spread of effective adaptations, to regional or global collective action. We introduce a comprehensive portal of data and models on cultural adaptation to climate change, and we outline ways forward.

This article is part of the theme issue ‘Climate change adaptation needs a science of culture’.

## 1. Introduction

The United Nations underscores that climate change adaptation—by which decision-makers usually mean change in response to or anticipation of the risks posed by climate change [1]—must become a priority *now* [2]. Some researchers and policymakers advocate ‘entirely new practices' and ‘transformative change,’ which usually entail technocratic solutions recommended by experts; however, we know little about whether these new practices actually reduce the risks important to communities, even over the short term [3,4]. What is needed are systematic data on which solutions, new or old, are effective at reducing risk for communities and persist across time [5]. Luckily, scientists working at the intersection of climate change and culture, often in close collaboration with communities, already have evidence for what has worked—and what has not—for humans past and present. This includes cautionary tales of the perils of top–down interventions introduced by organizations or policymakers without engaging communities in decision-making. However, this existing evidence often fails to reach organizations, policymakers and researchers. Climate change adaptation needs to embrace the science of culture: funding priorities should be set with adequate information about how communities *actually* adapt to climate change.

Here, we review key studies of cultural adaptation to climate change and their implications for future research and policy interventions. We begin with an overview of the scientific study of culture, then organize our review by scale: community-centred approaches (the micro level), population-level outcomes (meso level) and global-level perspectives (macro level). We wrap our discussion by introducing a data portal that incorporates data ranging from foraging returns to emissions, all pertinent to cultural adaptation to climate change. Along the way, we identify what researchers still need to know about climate change adaptation, highlight strategies for how to support communities on the frontlines, and explore how work done collaboratively with communities can do both.

## 2. Why is culture relevant to climate change adaptation?

Different disciplines and sectors variously define ‘culture,’ but regardless of how it is defined, most agree that culture impacts climate change adaptation—for example, by influencing the adoption, innovation and persistence of solutions (e.g. [1,4,6,7]). Here, we adopt one definition that is inclusive of many others: culture refers to information learned or adopted from other people that is stored in the mind [8]—information that often produces or affects things that exist outside the mind, like social relationships, societies and objects.

### (a) How culture affects climate change adaptation

Culture is often treated as static, a barrier to adaptation [9], but it can be a mediator of adaptation [10,11]—for example, as a predictor of adaptation motivation [12]. Importantly, culture *generates* candidate adaptations, ideas that have not yet reduced climate risk but may when put to the test [1,5,13]. Examples include agricultural techniques, migration, and even climate-related policy and funding. Because their livelihoods are entwined with climate, farmers are often at the forefront of climate change mitigation and adaptation, creating innovations that can reduce greenhouse gas emissions [14] or selectively adopting suggestions from organizations, policymakers and researchers based on what works locally [15]. Humans past and present have often used migration to respond to climate-related risks—although, thanks to borders and other restrictions, climate-related migration can be difficult today. Migration can be short-term, especially for groups reliant on mobile resources like fish or animal herds [16], or permanent, when climate shocks are large and/or recurrent (e.g. [17]). Climate-related policy and funding are also cultural, with fads and fashions in funding and prioritized projects impacting what candidate solutions are available to communities [18]. For example, investment in urban green spaces has exploded, but meta-analyses reveal that urban green spaces can have neutral or even negative impacts on heat, energy consumption and equity, depending on the dynamics of the communities where they are introduced [19,20]. Urban green spaces are candidate adaptations; their adaptiveness depends on specific local social and ecological dynamics [5,21].

**Figure 1.**
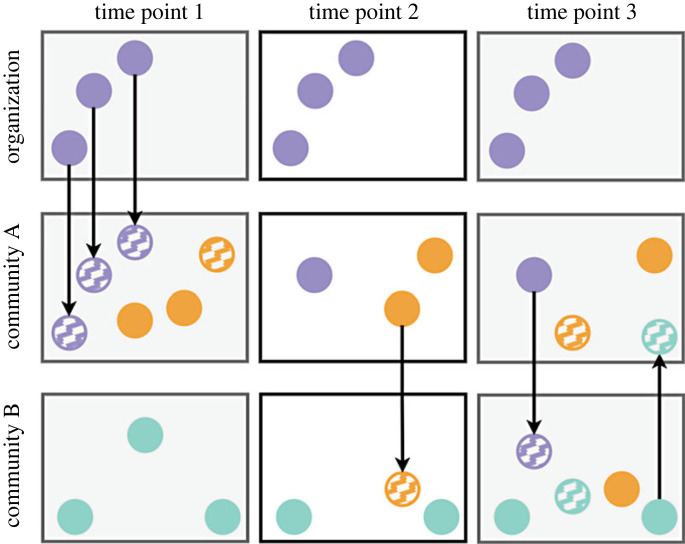
A review of the innovation and adoption, selective retention, and transmission (IARMT) of candidate climate change adaptations. For another overview in this issue, see [15]. At time point 1, an organization introduces candidate adaptations to community A (striped purple); A has existing local adaptations (solid orange) and a new innovation: a local *candidate* adaptation (striped orange). Nearby is community B, with their own local adaptations (solid green). At time point 2 in community A, in addition to one preexisting local adaptation, one introduced candidate adaptation and one local candidate adaptation were retained from time point 1; we now call these candidate adaptations ‘*adaptations’* (and use solid colours accordingly) because they persisted, presumably (although not always) because they reduce risk. Community B adopts a local adaptation from A; this is now a local *candidate* adaptation in B (striped orange) because it has not yet reduced risk locally. At time point 3, community B has retained the local candidate adaptation from A, making it a local adaptation in B (solid orange). Under ideal circumstances, the organization will learn and perhaps adopt local adaptations from A or B as well.

To illustrate the process of climate change adaptation, imagine two communities, A and B, and an outside organization whose mission it is to foster climate change adaptation (figure 1). Candidate adaptations are innovated—whether by community members or organization staff, usually built on or modifying existing variants. If candidate adaptations successfully reduce risk—that is, if they're adaptive—they are more likely to be retained and transmitted, whether to members of the same community or even across community boundaries (e.g. between A and B in figure 1). Retention and transmission happen (a) through memory, teaching and learning, and (b) for a variety of reasons, including cognitive biases (e.g. a candidate adaptation may be more memorable or more consistent with what is already in someone's mind) and how much candidate adaptations help their bearer respond to risks, including from climate [8,22–24]. For example, worldview impacts which innovations are adopted and which are ignored [9,25–29]. In turn, innovation and adoption, selective retention, modification and transmission are path-dependent, as past events or decisions constrain later events or decisions [30]. Innovation and adoption, selective retention, modification and transmission (IARMT) are processes with parallels in biological adaptation; indeed, because of these similarities, some use the phrase ‘cultural evolution’ to talk about how culture changes or stays the same across time (for reviews, see [15,31]).

### (b) *Studying* how culture affects climate change adaptation

The science of culture is not limited to the field of cultural evolution, however: research on the role of culture in climate change adaptation spans disciplines, sectors and methodologies. Psychologists and sociologists study the degree to which subjective internal states (e.g. values, perceptions, appraisals)—affected by culture among other things—impact behaviour related to climate change adaptation, including IARMT [9,32]. Indigenous and rural peoples have long innovated their own candidate adaptations. Indeed, Indigenous and Local Knowledge affects both internal states, like values and IARMT; further, while Indigenous and Local Knowledge is largely absent from earlier, Western research on climate change adaptation, work involving Indigenous methodologies, knowledge co-production, and participatory action frameworks is increasingly common in the adaptation literature [33–35]. For example, in their adaptation research in Samoa, Latai-Niusulu *et al*. [16] used *talanoa* methods—unstructured interviews in which stories are freely shared, inspired by Pacific Island communities. Historians and archaeologists sometimes track changes in environment and culture longitudinally, over centuries or millennia, by reconstructing paleoclimates and aggregate data from multiple archaeological sites (e.g. [36–40]). Hoyer *et al*. [17] integrate data on 150+ societies from the past 10 000 years to investigate which variables predict how societies respond to crisis. Cross-cultural ethnographic and survey data provide a synchronic view of the relationship between environment and culture, at the level of individuals and societies (e.g. [41–43]). Several articles in this theme issue provide detailed qualitative and quantitative data on the relationship between cultural practices and contemporary climate, which can be compared and contrasted across contexts [16,44–46] and can be compared to environmental data, such as carbon emissions data [14]. Agent-based models draw from both past and contemporary data to simulate future outcomes (e.g. [46–48]). In short, scientific approaches to culture take many forms, all with implications for our understanding of climate change adaptation.

## 3. What is known about culture and climate change adaptation?

Using diverse methods, scientists of culture have gained insight into climate change adaptation with implications for future research and policy interventions. Here, we review key findings from this literature, some of which feature in this theme issue. We flag notes of caution throughout, with recommendations for how to avoid similar issues in the future.

Scientists of culture usually focus on the micro, meso, or macro levels of cultural adaptation—as do organizations and policymakers. The IARMT of culture takes place on the micro, individual level, but the evolution of climate change adaptation is apparent at the meso level—for example, in the frequency of candidate solutions and their spread within and between populations [15,49,50]. Macro-level approaches to climate change adaptation incorporate multiple populations of people, generating insight that spans time—for example, which variables predicted successful adaptation in past societies—and space—for example, how patterns within- and across-populations impact regional and global outcomes today. An organization may focus on micro-level investment in the hope that it will scale up to meso- or macro-level change, or on the macro level in the hopes of changing the balance of power between the Global South and the Global North [51]. We thus organize our discussion by the micro, meso and macro levels to facilitate quick navigation by researchers, policymakers and organizations alike.

### (a) The micro level

Micro-level approaches to climate change adaptation reveal how IARMT unfold, while also allowing researchers to identify hurdles to grassroots climate change adaptation [5,15]. To understand the extent to which communities are impacted by climate change, and what other concerns may be more immediate to them, researchers can use research design and methods sensitive to local context and in collaboration with community partners when appropriate [16,52,53]. Data collection methods may involve community members in research and can identify constraints on locally led adaptation, yet data collection need not be time intensive: for busy researchers, Buffa *et al*. [54] outline how a small team can obtain community-centred data on constraints to adaptation in as few as two weeks.

Local context is key to weaving community-held expertise into understandings of grassroots climate change adaptation [55,56]. One form of such expertise is traditional ecological knowledge (TEK), ‘a cumulative body of knowledge, practice and belief, evolving by adaptive processes and handed down through generations by cultural transmission, about the relationship of living beings (including humans) with one another and with their environment’ [6] . TEK often encompasses adaptations to climate, including climate variability [57]. For example, in response to contemporary climate risks, cultural practices promote food security—such as mobility in Samoa or sharing country foods in Canada (e.g. fish, seal, caribou) [16,57–59]. When communities can innovate, modify and transmit candidate adaptations without outside interference, their adaptive capacity may be higher (e.g. [15,46,54]). In other words, communities may be better able to respond to climate change [60] because they can experiment with different solutions [5].

***Caution:*** While communities may mix TEK with ideas introduced by organizations or policymakers [6], non-local cultural variants can crowd out local variants [47,61–63] and offer solutions that increase rather than reduce risks salient to communities (e.g. [22,24]). In turn, decreased transmission of TEK may prevent younger generations from acquiring candidate adaptations [57,64]. Given that contemporary climate change can be rapid, hurdles to grassroots adaptation can impact its ability to keep pace [1]. To avoid displacing TEK and other local knowledge, organizations and policymakers may consider: (a) introducing non-local ideas and letting communities decide whether to adopt them—rather than letting non-local prerogatives or power be the ultimate deciding factor [65,66]; (b) avoiding policies that prevent the use of TEK, as such policies can hinder the transmission of TEK to future generations [62,67]; (c) fostering information-sharing networks, especially if existing networks have been displaced by government interventions, colonialism and other forces [5]; and (d) supporting relationship to place, as TEK is tuned to specific localities [13,46,53,62]. Organizations and policymakers may propose ‘transformative’ adaptations that represent a break from past practices [4,68], but when communities have agency to pick their own solutions, especially solutions not heavily curtailed by outside constraints [53], the solutions they choose—whether traditional, modified, non-local or a mixture—are more likely to be effective and persistent than other options [33,68–71].

Candidate adaptations that communities choose to use often reflect the conditions they face—climate-related and otherwise. ***Caution:*** When asked, communities on the frontlines of climate change do not always perceive the impacts measured by atmospheric scientists [72]. This may mean that adaptations are working well [73]. For example, among Yucatec Maya, investment in cash cropping, social network connections and economic diversification can mediate the relationship between climate risks and reports of good versus bad harvest years [45]. Other impacts on crops, finances and well-being, such as market forces or government policies, may be more salient than climate change because they have more day-to-day impact [72,74,75]. Indeed, even if communities feel threatened by climate change, market forces and government policies can hinder their ability to adopt new candidate adaptations: for Inuit, changes in sea ice as well as increased reliance on cash employment are shifting hunting preferences and the equipment needed for harvesting country foods [37], and many households cannot afford this new equipment [75].

### (b) The meso level

Meso-level analyses of climate change adaptation often examine regional or population-level outcomes—such as changes in the frequency of candidate adaptations, revealing cultural evolution; to group-level collective action; and human-environment integration. To understand how population structure aids or hinders climate change adaptation, consider a population with homophilous subpopulations that do not interact regularly *but* are open to learning from each other. Contexts like these can foster transmission of candidate adaptations [47,68,76]. Similarly important are majority–minority dynamics, e.g. between minoritized and majority ethnic groups, or between urban and rural communities. Even if subpopulations transmit cultural information to each other, if the flow of majority ideas to minority subpopulations is minimized—for example, if (a) minority subpopulation identity is strong, (b) the minority subpopulation has protected lands, or (c) organizations and policymakers refrain from swamping local solutions with non-local solutions—minority subpopulations can be important sources of innovation that may be adopted by the majority [47,77,78]. Per our recommendations above, if organizations and policymakers wish to encourage innovation and transmission in minority subpopulations, they can support relationship to place (e.g. by backing land or resource rights) and community agency by studying, identifying and selecting candidate adaptations [26,79,80].

Culture is also relevant to analysis of collective action around common-pool resources—resources that can be depleted and from which users are difficult to exclude. Common-pool resources are particularly sensitive to overuse: when individuals prioritize their private benefit over group outcomes—benefits that can be shared by all users—a tragedy of the commons can result [81]. However, individual and group benefits need not be at odds: when users expect joint benefit from cooperation or face the same outside threat (e.g. from pests or people from other user groups), collective action around common-pool resources is more likely [81–83]. Additionally, when individual and group interests are aligned, as may be the case with crop switching in the US [15], collective action is easier to get started. Organizations and policymakers wishing to foster collective action around common-pool resources can leverage groups and group dynamics---for example, by emphasizing shared threats or by encouraging groups to compete over who can best manage risk [7,82].

Scientists of culture also investigate interactions between human populations and the environment by focusing on the integration between them [84–86]. For example, consider rice paddies or swidden systems, also known as slash-and-burn, *milpa*, or shifting cultivation. Adjacent paddies and swiddens may become spatially correlated approximating a power law: a candidate adaptation adopted by one farmer impacts their neighbours in an exponentially decreasing, nonlinear way, such that close neighbours are more impacted than those further away [14]. This can signal a transition from exclusively local control to a complex adaptive system, which can approach Pareto optimality—where no one in the system could do better without negatively impacting someone else's outcomes [82]. Comparing and contrasting across socio-ecological systems has shown that specific recurrent features of the environment tend to favour similar cultural adaptations [17].

### (c) The macro level

Macro-level approaches to climate change adaptation incorporate multiple populations of people, allowing investigation of how individual- or population-level patterns can impact regional or global outcomes, and offering insight into past adaptation successes and failures from societies in human history and prehistory. Traditional forms of forest management offer an intriguing example. Humans have long used techniques like controlled burns and dispersing seeds to manage species in local forests [87,88]. Today, forests allow local communities to diversify their responses to climate risk—for example, to access alternative income streams, foods or sources of fuel (e.g. firewood). ***Caution:*** If policies remove other components of people's diversified risk-management portfolios, forests may become overharvested, reducing potential carbon stores and the intactness of traditional lands for Indigenous peoples [46]. Mitigation policies like REDD+ (Reduce Emissions from Deforestation and Forest Degradation) carbon credits, for example, can favour technocratic solutions for management that displace customary management systems; allowing some traditional management and limited harvesting can help protect people's livelihoods [89]. Another reason to support customary management systems is their potential impact on global outcomes: anthropogenic forests can respond differently to climate change than do unmanaged forests [88] and recognizing Indigenous territories has been shown to reduce deforestation inside their borders [90,91]; in other words, in some cases, adaptation and mitigation can be complementary.

In other cases, like the REDD+ example highlighted above, climate change mitigation and adaptation are sometimes at odds. For example, among Diné and Hopi peoples, data suggest that reduced access to coal, consistent with mitigation goals set by the government, will undercut energy sovereignty, key to climate change adaptation (e.g. staying warm in winter) [46]. At the global scale, mitigation efforts often emphasize energy efficiency. Paradoxically, as energy becomes more efficient, use may actually increase, reducing the potential energy savings of increased efficiency [92]. However, additional cultural innovation can offset these effects: if efficiency, or even carbon sequestration technology, increases faster than increased demand—if improvements in technology can be accelerated or reductions in demand be incentivized—this paradox may not be realized [48].

Analysis of paleoenvironmental, archaeological and historical data provides insight into past responses to climate stress [88,93,94]. Multi-population datasets can include indicators of social instability and other variables that impact population-level responses to climate events [17,95], as well as cascading risks where failures in one area of the world impacted others [96]. Unfortunately, insights on how past societies successfully responded to climate, or failed to do so, often do not reach policymakers [39,40,97]. For useful overviews of what is known about the effectiveness of different candidate adaptations in the face of myriad climate-related risks, see [37,38].

***Caution:*** Archaeological and historical data remind us that grassroots climate change adaptation does not always produce effective solutions. Because innovations often build on one another, path dependence can constrain adaptation—for example, given existing norms and practices in use [21,30,53]. Due to adaptive lag, variants that used to reduce risk may no longer be adaptive when the environment changes, but may still be retained and transmitted at higher rates because they are more memorable or more consistent with what is already in the mind [8,22,24]. That said, while local cultural variants are not *always* effective at reducing risk, they *can* be [21]—so rather than swamping communities with non-local candidate adaptations or, at the other extreme, leaving communities to adapt on their own, organizations and policymakers can support opportunities for local innovation [5,53]. Cultural analyses can help by offering insights on how climate change adaptation emerges and spreads [47] and, through active collaboration with communities, by identifying which local solutions are locally effective and persistent [54]. Climate justice involves both protecting communities on the frontlines and recognizing and supporting these communities' agency in selecting their own candidate climate change adaptations [65].

### (d) Cross-level interactions

Importantly, all three levels—micro, meso and macro—are interconnected and often overlapping. For example, the adoption of variants by individual farmers at the micro level (like different approaches to watering) affects ecology at the meso level [98] and determines which adaptations become locally predominant [14,15]. In other cases, national-level policies (macro) impact the transmission of TEK (micro), with implications for its preservation among Indigenous peoples (meso; [44,46]). In short, though scientists, organizations and policymakers may focus on just one of these three levels to make their research tractable or to set funding priorities, all three are fundamentally interconnected. For a pertinent review, see [99].

### (e) A portal for the curious

The data we summarize above range from household data, to archaeological data, to data on carbon emissions from rice paddies—all pertinent to the study of cultural adaptation to climate change. Curious readers can visit the Dryad Digital Repository for this theme issue: https://doi.org/10.5061/dryad.bnzs7h4h4 [100], where contributors have uploaded examples of these diverse data and often the code needed to analyze it, along with detailed readmes. Example uses of these data include: (a) comparing and contrasting field-based research on climate change adaptation—for example, exploring a potential negative relationship between the effectiveness of existing adaptations and the salience of local climate impacts; (b) modifying and exploring the predictions of models—for example, of how changes to social network characteristics change impact the innovation of candidate adaptations, or of how much incentive for cultural adaptation is needed to increase energy efficiency faster than demand; and (c) adopting and deploying tools used by researchers to study cultural adaptation to climate change, including community-collaborative research methods and leading-edge statistical models.

## 4. Conclusion

Recent reviews of the climate adaptation-related literature have concluded that little is known about the effectiveness and persistence of different climate change adaptations, largely due to a lack of longitudinal data [4]. However, outside the climate literature, scientists of culture have been studying the relationship between culture and environment for decades: for example, how innovative candidate adaptations reflect local experiences of climate, how population structure impacts the spread of adaptations and how these adaptations impact outcomes for other populations in turn. Going forward, scientists of culture must continue to reach out to organizations and policymakers to share what we have learned. Better understanding how climate change adaptation unfolds, at the micro, meso and macro levels, will enable organizations and policymakers to better support communities as they respond, by providing resources and respecting their autonomy [5].

## Data Availability

Data uploaded by issue contributors are available from the Dryad Digital Repository: https://doi.org/10.5061/dryad.bnzs7h4h4 [100].
